# Comparison of clinical outcomes of open and closed cardioplegia sets used during cardiopulmonary bypass

**DOI:** 10.1590/1806-9282.20250697

**Published:** 2025-10-17

**Authors:** Mahmut Padak, Bişar Amaç, Reşat Dikme, Murat Ersoy

**Affiliations:** 1 Harran University, Faculty of Health Sciences, Department of Perfusion – Şanlıurfa, Turkey.; 2 Harran University, Vocational School of Health Services, Dialysis Programme – Şanlıurfa, Turkey.; 3 New Hospital, Department of Cardiovascular Surgery – Şanlıurfa, Turkey.

**Keywords:** Cardiopulmonary bypass, Cardioplegia, Infection, Cardiac arrest

## Abstract

**OBJECTIVE:**

The aim of this retrospective study was to evaluate early clinical outcomes and inflammatory responses associated with the use of open and closed cardioplegia sets in patients undergoing cardiac surgery with cardiopulmonary bypass.

**METHODS:**

A total of 123 adult patients who underwent elective cardiac surgery with cardiopulmonary bypass between 2023 and 2024 were included. Patients were divided into two groups based on the cardioplegia system used: Group 1 (open system, n=63) and Group 2 (closed system, n=60). Demographic features, intraoperative variables, and preoperative and postoperative biochemical parameters (white blood cell count, C-reactive protein, glucose, and liver and renal function tests) were analyzed. Statistical significance was set at p<0.05.

**RESULTS:**

Preoperative characteristics and most laboratory parameters were comparable between the groups (p>0.05). Postoperatively, white blood cell (12.64±3.47 vs. 8.43±4.43, p=0.031), C-reactive protein (28.00±32.28 vs. 8.42±6.37, p<0.0001), glucose (150.66±82.40 vs. 174.19±51.89, p=0.043), aspartate aminotransferase (p=0.001), and gamma-glutamyl transferase (p<0.0001) levels were significantly lower in the closed system group. Extubation time was longer in the closed group (8.56±3.18 vs. 5.15±0.66 h, p<0.0001), while urine output was significantly lower (554.16±401.50 vs. 916.34±709.58 mL, p=0.001). No differences were observed in hospital or intensive care unit stay.

**CONCLUSION:**

Closed cardioplegia systems may offer better control of inflammatory and hepatic responses during cardiac surgery with cardiopulmonary bypass. Despite favorable biochemical outcomes, the longer extubation time and reduced urine output observed in the closed system group warrant further investigation. Future prospective randomized studies are required to validate these findings and assess long-term clinical outcomes such as morbidity and mortality.

## INTRODUCTION

Since the introduction of cardiopulmonary bypass (CPB), cardioprotective strategies have been continuously investigated to reduce ischemic damage to the heart during aortic cross-clamping (KAM) and reperfusion injury after unclamping. With the advances in cardiac surgery and percutaneous techniques, many cardiac surgical procedures can be performed with mechanical circulatory support. Therefore, there is an increasing need for advanced cardioprotective strategies to provide the best outcomes in patients undergoing cardiac surgery^1,2^.

Cardioplegic solutions have a very important place in cardiac surgery because they protect the myocardium against ischemic injury and ischemia-reperfusion injury by creating an immobile and bloodless working space^3^. The administration of a cardioplegic solution can increase the ischemic tolerance time in the heart up to several hours. Furthermore, CPB assumes the pumping function of the heart, providing oxygen and nutrients to organs and tissues and removing metabolites^4^.

In open systems, blood and crystalloid solutions are mixed in adjustable ratios using open tanks. Although this provides flexibility during surgery, it may increase thrombin generation by increasing the blood–air contact. Closed systems are designed as closed, compressible tanks and heparin-coated circuits. Closed systems minimize blood–air contact and reduce thrombin generation, thus reducing the risks of systemic inflammation and coagulopathy. Closed systems support single-dose delivery strategies with constant blood-to-crystalloid ratios and provide myocardial protection^5^.

Closed-system cardioplegia sets are applied with the help of a heart–lung machine. The heart–lung machine is a system that circulates blood through the body, provides oxygen, and removes carbon dioxide. This fluid is administered in conjunction with the heart–lung machine. During the operation, the heart–lung machine pumps blood to the patient and at the same time delivers cardioplegia solution to the heart. The fluid used to stop the heart usually contains potassium, magnesium, glucose, and other preservatives. This fluid is delivered to the heart through the pump of the heart–lung machine in a closed system.

Open-system cardioplegia sets are a system that allows the cardioplegia solution to be delivered to the heart under different pressure applications rather than through the KAM. While preparing the cardioplegia solution, first, the desired amount of oxygenated blood is drawn from the oxygenator through a cannula into the infusion bag, and drugs such as potassium and magnesium to be used to obtain cardioplegia are injected into it. After the necessary procedures, special cannulas or tubes are used to deliver this hypothermically prepared cardioplegia solution to the heart. These devices ensure that the fluid to reach the heart is delivered at the correct pressure and speed using a material such as a cuff (to create pressure).

The aim of this study was to evaluate the differences between the infection risks of open and closed cardioplegia systems and to determine the effects of these systems on surgical applications.

## METHODS

Patients with known systemic inflammatory diseases, pneumonia, chronic obstructive pulmonary disease (COPD), etc., before surgery were excluded from the study. After the exclusion criteria were applied, patients between the ages of 20 and 85 years who underwent cardiac surgery with CPB at the Cardiovascular Surgery Clinic of Harran University Hospital between 2023 and 2024 were included in the study. It was approved by the decision numbered 09 and 11, dated 01.07.2024, of the Clinical Research Ethics Committee of Harran University Faculty of Medicine. This study was conducted in accordance with the Declaration of Helsinki, as revised in 1989.

### Data collection method

Patient data were collected from electronic medical records, operating theater records, perfusion follow-up records, intensive care follow-up cards, and file records. All data were recorded and entered into the computer for analysis.

### Statistical analysis

Statistical analyses were performed using the SPSS^®^ 17.0 computer program (version 17.0, SPSS, Chicago, IL, USA). Means and standard deviations were calculated for continuous and ordinal data. The Kolmogorov-Smirnov test and Shapiro-Wilk test were used to assess the normality of distribution. Student's t-test and the Mann-Whitney U test were used to evaluate normally and non-normally distributed data, respectively. Frequency and percentage analyses were performed for nominal data, and the chi-square test was used for comparison. A p<0.05 was considered statistically significant.

## RESULTS

A total of 123 patients were included in this retrospective study. There were 63 patients in Group 1 and 60 patients in Group 2. The type of surgical operation, gender, age, height, weight, body surface area (BSA), and flow were similar in both groups (p=0.198, p=0.127, p=0.081, p=0.837, p=0.294, p=0.119, and p=0.062, respectively) (Table 1).

**Table 1:** Demographic characteristics of the groups.

Variables		Group 1 (mean±SD)	Group 2 (mean±SD)	p
Surgical type n (%)	AVR	6 (9.5%)	3 (5.0%)	0.198
	MVR	10 (15.9%)	8 (13.3%)	
	CABGX3	16 (25.4%)	17 (28.3%)	
	CABGX4	22 (34.9%)	15 (25.0%)	
	CABGX5	5 (7.9%)	11 (18.3%)	
	Aneurysm	4 (6.3%)	6 (10.0%)	
Gender n (%)	Female	17 (27%)	24 (40%)	0.127
	Male	46 (73%)	36 (60%)	
Age (years)		57.60±11.55	64.91±7.59	0.081
Height (cm)		167.30±8.48	170.31±9.41	0.837
Weight (kg)		77.44±13.56	73.08±15.55	0.294
BSA (m^2^)		1.85±0.16	1.77±0.24	0.119
Flow (L)		4.46±0.40	4.20±0.61	0.062

Mean±SD: Mean±standard deviation; BSA: body surface area; AVR: aortic valve replacement; MVR: mitral valve replacement; CABG: coronary artery bypass graft.

Among the intraoperative variables of the groups, cross-clamp time, total perfusion time, intensive care unit (ICU) stay, hospital stay, and temperature were similar (p=0.628, p=0.104, p=0.449, p=0.449, p=0.071, and p=0.329, respectively). However, there was a significant difference between the extubation time and urine volume of the groups (p<0.0001 and p=0.001, respectively) (Table 2).

**Table 2:** Intraoperative variables of the groups.

Variables	Group 1 (mean±SD)	Group 2 (mean±SD)	P
Cross-clamp duration (min)	59.74±28.39	62.53±24.05	0.628
Total perfusion time (min)	94.12±37.45	100.61±35.57	0.104
Extubation time (hours)	5.15±0.66	8.56±3.18	**<0.0001**
ICU time (days)	2.20±1.62	2.21±2.13	0.449
Length of hospital stay (days)	5.90±3.85	5.65±4.28	0.071
Urine volume (mL)	916.34±709.58	554.16±401.50	**0.001**
Temperature (°C)	31.98±0.12	32.00±0.00	0.329

Mean±SD: Mean±standard deviation; ICU: intensive care unit; SD: standard deviation. Statistically significant values are denoted in bold.

As shown in Table 3, preoperative data of the groups were similar (p>0.05). Postoperative urea, creatinine, potassium, alanine aminotransferase (ALT), lymphocyte, monocyte, neutrophil, and eosinophil levels were also similar (p>0.05). However, there was a significant difference between the postoperative white blood cell (WBC), C-reactive protein (CRP), and glucose levels of the groups (p=0.031, p<0.0001, and p=0.043, respectively). There was also a significant relationship with the presence of a negative correlation in the postoperative aspartate aminotransferase (AST) and gamma-glutamyl transferase (GGT) levels of the groups (p=0.001 and p<0.0001, respectively).

**Table 3:** Comparison of preoperative and postoperative data of the groups.

Variables	Group 1 (mean±SD)	Group 2 (mean±SD)		p
Preoperative WBC (mm^3^)	9.57±2.68	6.86±2.83		0.368
Postoperative WBC (mm^3^)	12.64±3.47	8.43±4.43		**0.031**
Preoperative CRP (mg/L)	1.98±3.26	2.61±2.25		0.653
Postoperative CRP (mg/L)	28.00±32.28	8.42±6.37		**<0.0001**
Preoperative glucose (mmol/L)	168.90±49.89	187.59±57.27		0.223
Postoperative glucose (mmol/L)	150.66±82.40	174.19±51.89		**0.043**
	**(Mean rank)**	**(Mean rank)**	**Z**	**p**
Preoperative urea (mg/dL)	56.98	67.27	-1.599	0.110
Postoperative urea (mg/dL)	61.33	62.70	-0.213	0.832
Preoperative creatine (mg/dL)	62.89	61.07	-0.283	0.777
Postoperative creatine (mg/dL)	65.53	58.29	-1.126	0.260
Preoperative potassium (mmol)	58.68	65.48	-1.058	0.290
Postoperative potassium (mmol)	63.70	60.22	-0.542	0.588
Preoperative AST (U/L)	56.22	68.07	-1.842	0.065
Postoperative AST (U/L)	72.88	50.58	-3.469	**0.001**
Preoperative ALT (U/L)	58.15	66.04	-1.227	0.220
Postoperative ALT (U/L)	68.01	55.69	-1.915	0.055
Preoperative GGT (U/L)	59.66	64.46	-0.747	0.455
Postoperative GGT (U/L)	75.71	47.61	-4.371	**<0.0001**
Preoperative lymphocyte (μL)	61.88	62.13	-0.038	0.970
Postoperative lymphocyte (μL)	59.24	64.90	-0.880	0.379
Preoperative monocyte (%)	60.78	63.28	-0.390	0.697
Postoperative monocyte (%)	60.32	63.77	-0.536	0.592
Preoperative neutrophil (μL)	59.60	64.53	-0.767	0.443
Postoperative neutrophil (μL)	60.67	63.40	-0.425	0.671
Preoperative eosinophil (cells/μL)	59.88	64.23	-0.676	0.499
Postoperative eosinophil (cells/μL)	56.25	68.04	-1.838	0.066

Mean±SD: Mean±standard deviation; WBC: white blood cell; CRP: C-reactive protein; AST: aspartate aminotransferase; ALT: alanine aminotransferase; GGT: gamma-glutamyl transferase. Statistically significant values are denoted in bold.

## DISCUSSION

Infections following both open and closed CPB operations pose significant challenges due to their potential to cause serious complications and affect patient outcomes. Causes of CPB infections include equipment-associated pathogens, prolonged operative times, open systems, and patient-specific factors such as hyperglycemia^6-8^.

When comparing open and closed CPB systems, the focus is usually on their impact on infection rates and related complications. Closed CPB systems are generally designed to reduce complications by minimizing the blood–air interface and improving biocompatibility. Closed CPB systems (especially heparin-coated systems) have been shown to reduce inflammatory markers such as complement factors and proinflammatory cytokines compared to conventional open systems. This suggests a potentially lower risk of inflammation-related complications that may contribute to infections. Studies suggest that closed systems may lead to reduced coagulation and fibrinolytic activation, which may reduce postoperative bleeding and the need for blood transfusions and indirectly reduce the risk of infection^9,10^. This suggests a potentially lower risk of inflammation-related complications that may contribute to infections.

Closed cardioplegia systems have been linked to improved maintenance of cerebral tissue oxygenation and a reduction in microembolization events, factors which may collectively contribute to a lower incidence of neurological complications and thereby indirectly decrease the risk of postoperative infections^11^. Furthermore, the use of closed systems has been associated with reduced postoperative bleeding and a diminished need for allogeneic blood transfusions, which could potentially lower infection risk by limiting exposure to exogenous pathogens^10,11^.

However, despite these theoretical and clinical advantages, several studies have reported no significant differences between open and closed systems in terms of coagulation profiles, inflammatory responses, or overall biocompatibility, implying that the type of cardioplegia delivery system employed may not substantially impact postoperative infection rates^12^.

In this retrospective study, we evaluated the effects of open and closed cardioplegia systems on early postoperative outcomes. The results are discussed below in a structured manner:

### Postoperative inflammatory response (white blood cell and C-reactive protein)

Postoperative WBC and CRP levels were significantly higher in the open system group (p=0.031 and p<0.0001, respectively). These findings suggest that the open cardioplegia system may trigger a more intense systemic inflammatory response, potentially due to greater blood–air exposure and lack of surface coating. Prior studies have reported similar observations, indicating that closed, heparin-coated systems can attenuate systemic inflammation^9,10^.

### Postoperative glucose levels

Postoperative glucose levels were significantly elevated in the open group (p=0.043). Stress-induced hyperglycemia is commonly observed after CPB and is known to exacerbate inflammatory processes and increase infection risk^8^. The elevated glucose levels in the open group may reflect heightened inflammatory and stress responses.

### Liver enzymes (aspartate aminotransferase and gamma-glutamyl transferase)

Postoperative AST and GGT levels were significantly lower in the closed group (p=0.001 and p<0.0001, respectively), suggesting better preservation of hepatic function. Although alanine aminotransferase (ALT) levels did not reach statistical significance (p=0.055), they followed a similar trend. This may indicate a potential hepatoprotective effect of the closed system by reducing oxidative and inflammatory stress on hepatic tissue.

### Extubation time

Extubation time was significantly longer in the closed system group (p<0.0001). This finding may appear contradictory given the reduced inflammatory markers. Possible explanations include differences in myocardial metabolic response, anesthetic clearance, or fluid overload. Further studies are warranted to clarify the clinical relevance of this parameter.

Although closed cardioplegia systems were associated with reduced inflammatory and hepatic markers, the extubation time was significantly longer in this group. This finding may be attributed to several factors, such as intraoperative fluid management, myocardial metabolic recovery dynamics, or anesthetic clearance rates. It is also possible that a more conservative weaning approach was adopted in patients with closed systems due to lower urine output or individual perfusion parameters. Despite the lower levels of inflammatory markers observed in the closed-system group, the significantly prolonged extubation time may be attributed to intraoperative fluid balance, myocardial metabolic recovery, or conservative ventilation protocols—all of which warrant further investigation. These hypotheses should be explored in future prospective studies specifically designed to monitor cardiopulmonary and respiratory parameters in greater detail.

### Urine output

The open-system group showed significantly higher urine output postoperatively (p=0.001). While this may indicate better renal perfusion, it could also reflect differences in intraoperative fluid management or diuretic use. Closed systems may reduce systemic inflammatory response but may also influence renal hemodynamics. The long-term renal implications of this finding remain to be elucidated.

The finding of higher urine output in the open group may reflect better renal perfusion or different fluid administration protocols. One explanation could be increased preload or systemic pressure fluctuations related to the open system's exposure to atmospheric pressure. Moreover, hemodynamic differences or variations in diuretic use may have contributed to this difference. Although renal function markers (urea and creatinine) were not significantly different between the groups, this finding warrants further investigation in studies that include intraoperative renal perfusion monitoring. The increased urine output observed in the open-system group may reflect differences in fluid administration or diuretic use, rather than superior renal function, as no significant differences in creatinine or urea levels were detected.

### Other parameters

No significant differences were observed between the groups in terms of postoperative urea, creatinine, potassium, ALT, lymphocyte, monocyte, neutrophil, and eosinophil levels, ICU stay, or hospital stay. These findings suggest that while the closed system may modulate specific inflammatory and hepatic responses, it does not drastically affect general organ function or clinical recovery time within the early postoperative period.

Innovations in closed cardioplegia delivery systems offer versatility and safety by enabling rapid adaptation to different cardioplegia strategies without compromising sterility. This system supports multiple cardioplegia solutions and reduces the risk of perfusion accidents, thus improving the efficiency and safety of cardiac surgeries^13^. The design of closed circuits also contributes to reduced hemostatic activation by minimizing blood–air interface and avoiding cardiotomy suction^14^. Such strategies are essential in reducing postoperative coagulation-related complications.

The degree of hemostatic activation during CPB can be influenced by the design of the perfusion system. Closed systems that minimize blood–air interface and prevent cardiotomy suction have been shown to significantly reduce hemostatic activation. This is achieved by using passive ventilation techniques that further seal the system and reduce blood exposure to foreign surfaces^14^. Such strategies are crucial in minimizing coagulation-related complications during and after surgery.

Although closed cardioplegia systems are associated with several theoretical and physiological advantages—such as improved tissue oxygenation, reduced microembolization, and decreased need for allogeneic transfusions—current clinical evidence has not yet definitively demonstrated superior outcomes in terms of morbidity and mortality^9,10,15^. While some studies report favorable effects on surrogate markers, including biochemical indicators of inflammation and hemodynamic stability, these findings have not been consistently translated into meaningful differences in long-term survival, major adverse cardiac and cerebrovascular events, or hospital readmission rates^9,10^.

Moreover, the existing literature largely consists of single-center, retrospective studies with limited sample sizes, making it difficult to generalize the results or draw firm conclusions about the clinical superiority of closed systems^15^. To overcome these limitations, there is a critical need for well-designed, large-scale, prospective, and randomized controlled trials involving multiple surgical centers and standardized protocols. Such studies are essential to determine whether the physiological benefits observed with closed cardioplegia techniques can be reliably linked to improved patient-centered outcomes across diverse populations and clinical settings^15^.

## CONCLUSION

In this study, closed cardioplegia sets demonstrated beneficial effects on early postoperative outcomes compared to open systems. Although no significant differences were found in preoperative characteristics or most postoperative biochemical markers, closed systems were associated with significantly lower levels of postoperative WBC, CRP, and liver enzymes (AST and GGT), indicating a reduced inflammatory and hepatic response. Additionally, longer extubation times and reduced urine output were observed in the closed group, underscoring the need for further investigation into renal perfusion and respiratory recovery parameters. While these findings support the potential physiological benefits of closed systems, their clinical superiority cannot be definitively established based on retrospective data alone. Therefore, prospective, randomized studies are required to determine whether these early biochemical advantages translate into improved long-term outcomes in terms of morbidity and mortality.

## Data Availability

The datasets generated and/or analyzed during the current study are available from the corresponding author upon reasonable request.
